# Immunohistochemical expression of Stomatin-Like protein 2 and trophoblast cell surface antigen 2 in papillary thyroid carcinoma in comparison to other thyroid lesions

**DOI:** 10.1186/s13000-026-01772-0

**Published:** 2026-03-17

**Authors:** Ahmed A. Elmetwally, Marwa H. Khashaba, Heba M. Wagih, Azza H. M. Zidan, Mayada S. Farrag

**Affiliations:** 1 Department of Pathology, Faculty of Medicine, Port Said University, Port Said, Egypt; 2 Pathology Laboratory, Ismailia Teaching Oncology Hospital, Ismailia, Egypt; 3 Department of Pathology, Faculty of Medicine, Suez-Canal University, Ismailia, Egypt; 4 Department of Pathology, Faculty of Medicine, Galala University, Suez, Egypt

**Keywords:** SLP-2, TROP-2, PTC, Other thyroid lesions

## Abstract

**Background:**

Thyroid carcinoma is a common tumor affecting the Egyptian population accounting for 2% of newly diagnosed cases of cancer in Egypt yearly and constituting 7.73% of 5 year prevalence in Egypt. Stomatin-Like Protein 2 (SLP-2) and Trophoblast Cell Surface Antigen 2 (TROP-2) antibodies are both expressed in some types of thyroid carcinomas.

**Aim:**

To evaluate immunohistochemical expression of SLP-2 and TROP-2 in papillary thyroid carcinoma and other thyroid lesions, assess association of both immunohistochemical markers with variable demographic and histopathological parameters and assess the diagnostic utility of both markers in thyroid carcinomas.

**Subjects and methods:**

The current study is a cross-sectional study with analytical component, performed in the Pathology laboratory of Suez Canal University Hospital on 144 samples of formalin fixed paraffin embedded blocks of thyroid cases, including 79 cases of PTC and 65 cases of other thyroid lesions during the period from January 2016 to May 2019.

**Results:**

SLP-2 and TROP-2 have shown positive expression in 91.1% and 82.3% respectively in PTC cases. Compared to positive expressions in 38.5% and 7.7% respectively in other thyroid cases. The positivity of both markers has a statistically significant association with PTC cases, compared to other thyroid lesions p-value < 0.05). The sensitivity of combined positively expressed markers is 79.75%, specificity is 98.46%, positive predictive value is 98.44, the negative predictive value is 80% and the test accuracy is raised to 88.19 for discrimination between PTC and other thyroid lesions. There was no statistically significant association between all histopathological parameters and the expression of SLP-2 and TROP-2.

**Conclusions:**

SLP-2 and TROP-2 may be promising diagnostic markers of PTC carcinoma. Further studies are needed to fully evaluate the prognostic significance of SLP-2 and TROP-2 expression in PTC and other thyroid lesions.

## Introduction

Thyroid carcinoma is a common tumor affecting the Egyptian population accounting for 2% of newly diagnosed cases of cancer in Egypt yearly and constituting 7.73% of 5 year prevalence in Egypt. Thyroid carcinoma represents 3% of worldwide newly diagnosed cases yearly [1].

In some cases, there is difficulty reaching the final diagnosis, either by FNAC or even by examination of excision specimens. Second pathological opinion and molecular analysis could be needed in such cases [2].

The currently used immunohistochemical cocktail markers that aid in diagnosis of difficult cases are HBME1, CK19 and Galactin 3, but till now no single marker is diagnostic and their accuracy is still controversial [3].

SLP-2 is a mitochondrial membrane protein encoded by stomatin-homologous gene, SLP-2 protein has a role in regulation of proliferation, migration and invasion of many tumor cell types [4, 5]. Overexpression of SLP-2 inhibits phosphorylation of MAPK that induces MAPK pathway, resulting in tumor cells proliferation, migration and angiogenesis [6].

SLP-2 is overexpressed in many tumors including ovarian tumors, gastric cancer, colonic cancer, pancreatic cancer, cervical cancer, head and neck and esophageal squamous cell carcinomas [7, 8].

TROP-2 is a transmembrane glycoprotein encoded by tumor associated calcium signal transducer 2 (TACSTD2) gene [9]. It is over-expressed in many carcinomas as breast, pancreatic, cervical, lung, colorectal, gastric and ovarian cancers compared to normal tissues [10]. TROP-2 overexpression affects cell cycle and cellular proliferation by activation of TROP-2 protein domains leads to increased influx of cytoplasmic calcium influencing cell cycle activation and progression [11].

Also, due to presence of a phosphatidylinositol bisphosphate binding sequence at its cytoplasmic tail, it can be phosphorylated by protein kinases, leading to activation of mitogen-activated protein kinases (MAPKs), causing promotion of tumor growth and increased cell proliferation [12].

Some studies showed overexpression of SLP-2 and TROP-2 in papillary thyroid carcinomas [6, 13]. However, levels of expression of both SLP-2 and TROP-2 in different thyroid non-neoplastic and neoplastic lesions should be studied to evaluate their diagnostic accuracy and their association with other clinical and histopathological parameters.

## Materials and methods

### Ethical approval

The study protocol was reviewed and accepted by the (IRB/IEC) institutional review board and institutional research ethics committee of PSU prior to initiation (ERN: MED (1/6/2023) s.no (95) PTH 904_001).

The research involved no risks over researcher, sample or environment.

The need for informed consent has been waived by the (IRB/IEC) institutional review board and institutional research ethics committee of PSU.

The study used human data and material (paraffin blocks) and it was adherent to the declaration of Helsinki and this was insured by (IRB/IEC) institutional review board and institutional research ethics committee of PSU.

### Study setting and study population

The current study is a cross-sectional analytical study, performed in the Pathology laboratory of Suez Canal University Hospital on 144 cases, including 79 cases of PTC and 65 cases of other thyroid lesions, using computer based randomized sampling. Materials were collected from histopathological data and formalin fixed paraffin embedded blocks of thyroid cases available at Suez Canal University Hospital during the period from January 2016 to May 2019. Demographic and histopathological data includes gender, age at time of excision, tumor size, perineural invasion, lymphovascular invasion, tumor capsular invasion, extrathyroidal extension, lymph node metastasis and tumor stage.

### Inclusion criteria

1. Adequate samples that are finally diagnosed as papillary thyroid carcinoma and other thyroid non-neoplastic and neoplastic lesions, either benign or malignant.
2. Samples with available demographic and histopathological data.

### Exclusion criteria

1. Samples with scanty material which is not sufficient for proper immunohistochemical staining.
2. Cell blocks of FNAC specimens.

### Histopathology and immunohistochemistry procedure

Sections have been cut at 5 μm thickness. They have been stained using hematoxylin and eosin. Then they have been reviewed by the researcher and supervisors to classify the tumor type based on the latest WHO classifications of endocrine neoplasms [14].

Staging of the tumor was based on TNM system according to AJCC, eighth edition [15], followed by assessment of the presence of perineural invasion, lymphovascular invasion, tumor capsular invasion, extra-thyroidal extension and lymph nodal metastasis [14]. Block of tissue microarray have been prepared by manual extraction of areas of interest from the donor paraffin blocks, the extracted area has surface area of 25 mm^2^, each recipient block contains 15 samples, including 13 samples of areas of interest, in addition to a positive control for each of SLP-2 and TROP-2, Positive control of SLP-2 are sections from fallopian tube tissue and positive control of TROP-2 are sections from ectocervix. Negative controls are sections of the same specimens with omission of the primary antibody [16].

Sections have been cut at 5 μm thickness from each block and mounted on positively charged slides, followed by automatic staining of SLP-2 and TROP-2 using Ventana BenchMark GX device. Both the mouse monoclonal IgG purified SLP-2 catalogue number (sc-376165) of Santa Cruz biotechnology, Inc and TROP-2 catalogue number (sc-376181) of Santa Cruz biotechnology, Inc receptor antibodies at a dilution of 1/50 for 30 min have been used according to the protocol of use mentioned in the company data sheet.

### Evaluation and interpretation of immunostaining

Immunohistochemically stained slides were examined with a light microscope (Olympus BX53) for evaluation of immunohistochemical expression of SLP-2 and TROP-2 in tumor cells.

### Evaluation of SLP-2 immunostaining

Expression of SLP-2 will be evaluated for both proportion and intensity of staining. Slides will be considered as positive for SLP-2 Receptor expression if more than 10% of tumor cells show remarkable cytoplasmic staining [17].

### Evaluation of TROP-2 immunostaining

Expression of TROP-2 will be evaluated for both proportion and intensity of staining. Slides will be considered as positive for TROP-2 Receptor expression if more than 10% of tumor cells show remarkable membranous staining [17].

### Sample size calculation

The sample size was calculated using the following formula: n = k* s2 / d2 [18]. K = 2(Z1-α/2 + Z1-β)2, where Z1-α/2 = 1.96 for 95% confidence interval and Z1-β = 0.84 for an 80% power of study. S =expected standard deviation of the fold change of SLP2 or TROP-2 expression and D = effect size for expression of SLP2 or TROP-2 [17]. Accordingly, the calculated sample size for SLP-2 expression was 100 and the calculation for TROP-2 yielded a larger sample size of at least 128. Therefore, the larger sample size has been used at least in this study.

### Statistical analysis

Descriptive analysis will be done by calculating the percentage of different studied groups. Association between levels of SLP-2 and TROP-2 expression and different demographic and histopathological parameters will be assessed using the appropriate statistical tests according to types of variables. Visual display of results will be done using tables, charts and diagrams. p- value will be considered significant when < 0.05. Statistical analysis will be done using the statistics software SPSS (Version 21) for windows 10.

## Results

### Histopathological and demographic characteristics

**Fig. 1:**
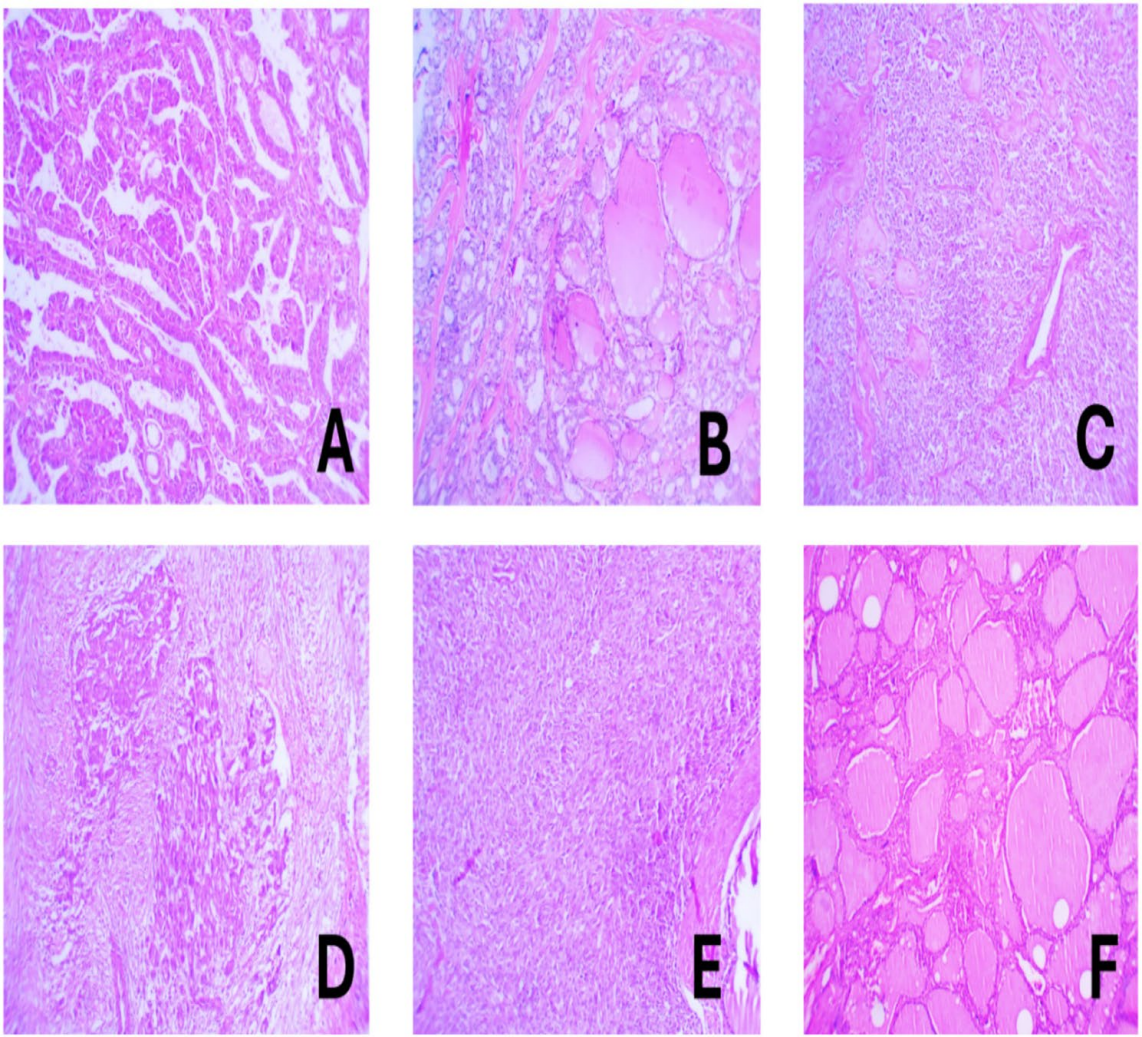
(**A**) Papillary thyroid carcinoma. Complex papillary architecture with fibrovascular cores, tumor cells are enlarged, oval, optically clear and overlapping (H&E, 100x). (**B**) Invasive follicular variant of papillary thyroid carcinoma. Tumor cells are arranged in follicles infiltrating surrounding thyroid tissue. Nuclei of tumor cells are enlarged, oval, overlapping and clearing (H&E, 100x). (**C**) Anaplastic thyroid carcinoma. Tumor cells are arranged in solid sheets, separated by thin fibrous stroma with evidence of tumor necrosis. Tumor cells show marked pleomorphism with hyperchromatic nuclei (H&E, 100x). (**D**) Medullary thyroid carcinoma. Tumor cells are arranged in solid sheets and nests, separated by thin fibrous stroma and showing areas of eosinophilic amyloid material, tumor cells are rounded and oval, some cells show eccentric nuclei with salt and pepper chromatin (H&E, 100x). (**E**) Follicular thyroid carcinoma. Tumor cells are arranged in solid sheets and infiltrating overlying thick capsule. Tumor cells are rounded with hyperchromatic nuclei, tumor cells show capsular invasion (H&E, 100x). (**F**) Follicular nodular disease, formed of multiple follicles, lined by uniform follicular epithelial cells and filled with colloid material (H&E, 100x)

This study was done on 144 cases of thyroid lesions; 79 cases are of papillary thyroid carcinomas and 65 cases were of other thyroid lesions, they were further subclassified into 19 non-neoplastic cases, 21 benign cases and 25 cases of other malignant tumors, including follicular thyroid carcinomas (9 cases), medullary thyroid carcinomas (11 cases) and anaplastic thyroid carcinomas (5 cases) (Table 1 and Fig. 1). The demographic and histopathological data of these cases are listed in (Tables 2 and 3).

**Table 1:** Distribution of PTC and other thyroid lesions according to specific subtypes

Groups	Nature	Diagnosis	No.	%
PTC (*n* = 79)		Papillary thyroid carcinoma	79	100.0
Other thyroid lesions (*n* = 65)	Non-neoplastic lesions	Follicular nodular disease	6	9.2
		Toxic goiter (Gravis)	7	10.8
		Hashimoto’s thyroiditis	6	9.2
	Benign lesions	Adenoma	21	32.3
	Other malignant lesions	Anaplastic thyroid carcinoma	5	7.7
		Follicular thyroid carcinoma	9	13.8
		Medullary thyroid carcinoma	11	16.9

**Table 2:** Comparison between PTC and other thyroid lesions according to demographic data (*n* = 144)

Variables	Categories	Other thyroid lesions (*n* = 65)		PTC (*n* = 79)		χ^2^	p
		No.	%	No.	%		
Age	Less than 55	42	64.6	64	81.0	4.936^*^	**0.026** ^*^
	55 or more	23	35.4	15	19.0		
Gender	Male	11	16.9	21	26.6	1.925	0.165
	Female	54	83.1	58	73.4		

χ^2^: Chi square test

*p*: *p* value for comparing the two studied groups

* Statistically significant at *p* ≤ 0.05

The bold is the statistically significant results

**Table 3:** Distribution of cases of PTC and other malignant thyroid lesions according to variable histopathological parameters (*n* = 104)

Variables		Other malignant thyroid lesions (*n* = 25)		PTC (*n* = 79)		χ^2^	p
		No.	%	No.	%		
Perineural invasion state	No	20	80.0	74	93.7	**χ²=** **4.084** ^*^	**0.043** ^*^
	Yes	5	20.0	5	6.3		
Lymphovascular invasion state	No	11	44.0	61	77.2	**χ²=** **9.835** ^*^	**0.002** ^*^
	Yes	14	56.0	18	22.8		
Capsular invasion	No	14	56.0	55	69.6	χ²= 1.578	0.209
	Yes	11	44.0	24	30.4		
Extra thyroid extension	No	20	80.0	57	72.2	χ²= 0.609	0.435
	Yes	5	20.0	22	27.8		
T stage	T1a	0	0.0	14	17.7	**FET = ** **13.342** ^*^	**0.012** ^*^
	T1b	2	8.0	10	12.7		
	T2	1	4.0	15	19.0		
	T3a	11	44.0	18	22.8		
	T3b	11	44.0	20	25.3		
	T4a	0	0.0	2	2.5		
Lymph nodal invasion status	Nx/0	21	84.0	61	77.2	χ²= 0.524	0.469
	N1	4	16.0	18	22.8		
Stage	I	5	20.0	68	86.1	**FET=** **43.172** ^*^	**< 0.001** ^*^
	II	15	60.0	9	11.4		
	III	0	0.0	2	2.5		
	IV	5	20.0	0	0.0		

*χ*^2^ Chi square test, *FET* Fisher Exact test

*p*: *p* value for comparing the two studied groups

^*^ Statistically significant at *p* ≤ 0.05

The bold is the statistically significant results

The age of cases ranged from 17 to 72 years, with a mean of 42.5 ± 15 years. Thirty-eight cases were ≥ 55 years old and 106 cases were less than 55 years old. There are 106 female cases and 38 male cases. 81% of PTC cases are < 55 years old, while only 64.6% of other thyroid lesions are < 55 years old. There is a statistically significant association between PTC and younger cases’ age (*p* = 0.026) (Table 2). There was no statistically significant association between the gender of cases and the diagnosis of thyroid lesions (Table 2).

Cases of other malignant thyroid tumors show stronger statistically significant association with perineural invasion, lymphovascular invasion, higher T stage and higher tumor stage than cases of papillary thyroid carcinomas (*p* ≤ 0.05) (Table 3).

Concerning capsular invasion, extra-thyroid extension and lymph nodal invasion status there were no statistically significant differences between cases of papillary thyroid carcinoma and other malignant thyroid cases (Table 3).

### SLP-2 immunohistochemical expression

**Fig. 2:**
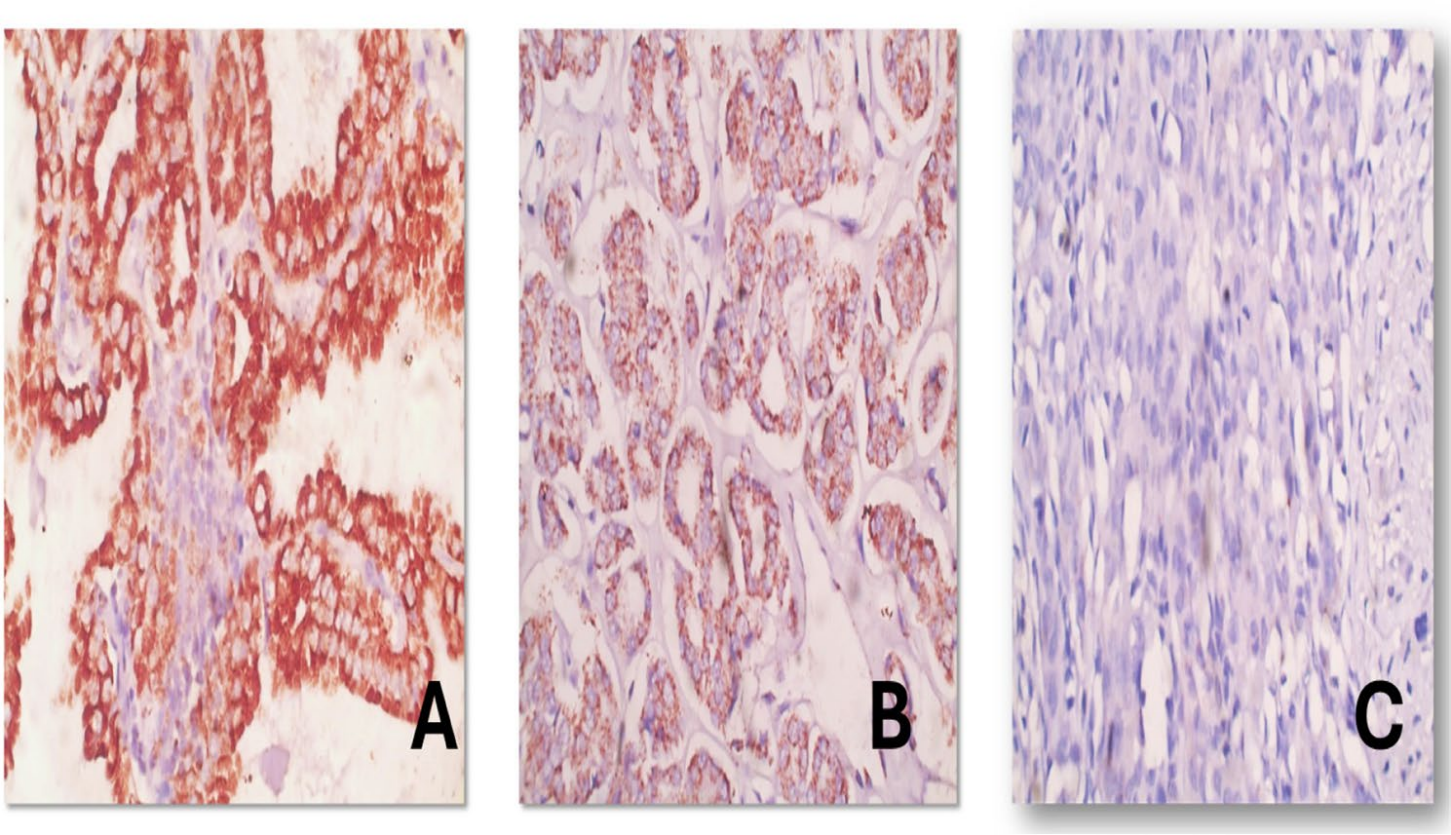
(**A**) Strong pvositive cytoplasmic immunohistochemical expression of SLP-2 in 90% of tumor cells in a case of papillary thyroid carcinoma (SLP-2, DAB, hematoxylin, 400x). (**B**) Moderately positive cytoplasmic immunohistochemical expression of SLP-2 in 70% of tumor cells in a case of follicular variant of papillary thyroid carcinoma (SLP-2, DAB, hematoxylin, 400x). (**C**) Negative immunohistochemical expression of SLP-2 in tumor cells in a case of anaplastic thyroid carcinoma (SLP-2, DAB, hematoxylin, 400x)

Cases were immunohistochemically stained for SLP-2, ninety-seven cases were positive for SLP-2, representing 67.4% of all cases. Fifty cases showed strong SLP-2 expression, 54 cases showed moderate SLP-2 expression, eight cases showed faint non conclusive expression of SLP-2, and 32 cases showed no SLP-2 expression **(**Fig. 2). Seven cases of showing a moderate SLP-2 intensity were interrupted as negative, hence they showed a proportion of staining of less than 10%.

**Fig. 3:**
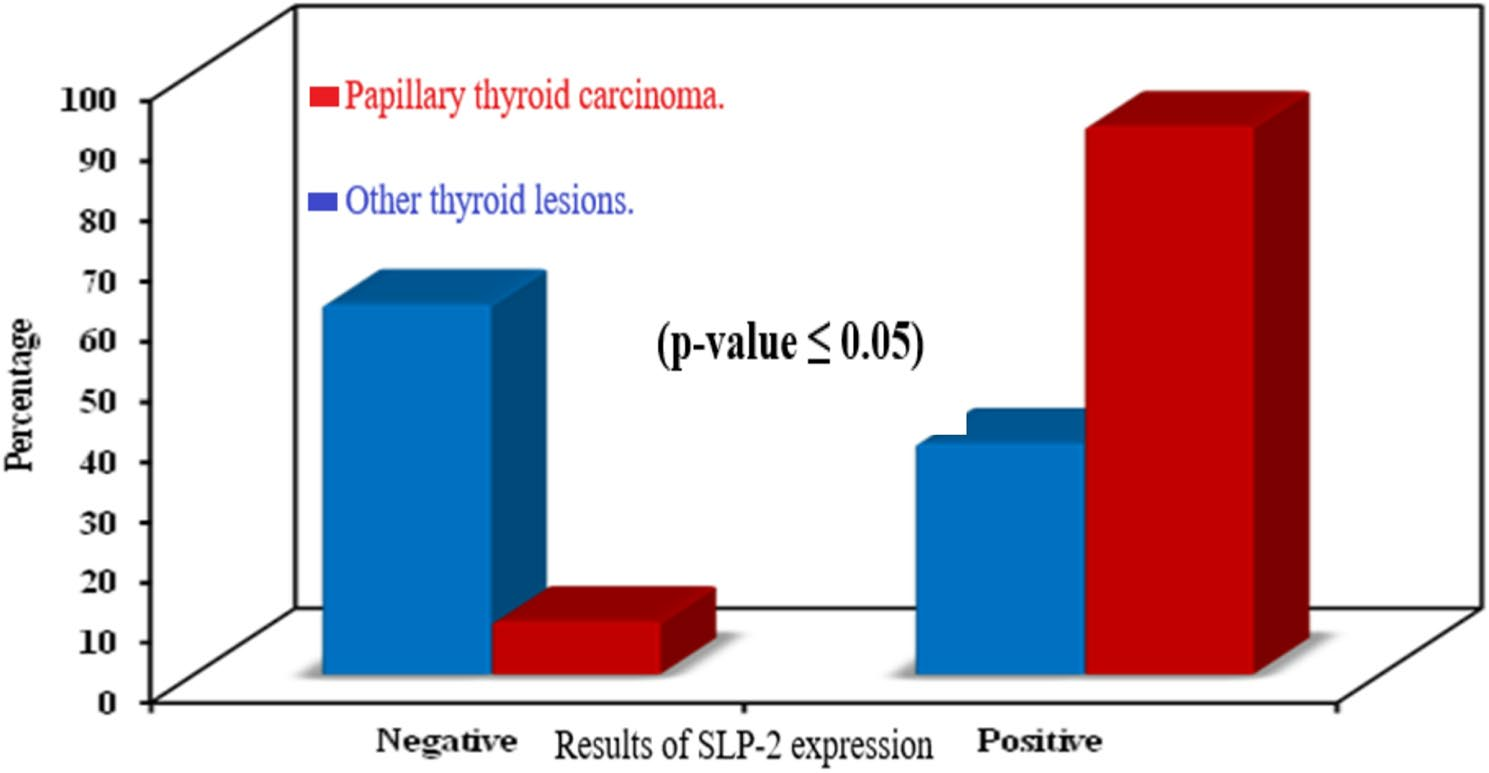
Distribution of cases of PTC and other thyroid lesions according to results of SLP-2 expression

Seventy-two cases of papillary thyroid carcinoma were positive for SLP-2, representing 91.1% of group cases. 25 cases of other thyroid lesions out of 65 were positive for SLP-2, representing 38.5% of group cases. Papillary thyroid carcinoma samples have shown a statistically significant association with positive expression for SLP-2 (*p* ˂0.001) (Fig. 3). Also, samples of papillary thyroid carcinoma have a statistically significant association with stronger expression of SLP-2 than other thyroid cases (*p* ˂0.001) (Table 4).

**Table 4:** Distribution of cases of PTC, other thyroid lesions and other malignant tumors according to results of SLP-2 expression (*n* = 144 & 104 respectively)

SLP-2 expression		Other thyroid lesions (*n* = 65)		PTC (*n* = 79)		*p*-value
		No.	%	No.	%	
Intensity	Negative	26	40.0	6	7.6	**< 0.001** ^*^
	Faint	8	12.3	0	0.0	
	Moderate	24	36.9	30	38.0	
	Strong	7	10.8	43	54.4	
Result	Negative	40	61.5	7	8.9	**< 0.001** ^*^
	Positive	25	38.5	72	91.1	

SLP-2 expression		**Other malignant tumors (** ***n*** ** = 25)**		**PTC** **(** ***n*** ** = 79)**		***p*** **-value**
		**No.**	**%**	**No.**	**%**	
Intensity	Negative	8	32.0	6	7.6	**< 0.001** ^*^
	Faint	2	8.0	0	0.0	
	Moderate	12	48.0	30	38.0	
	Strong	3	12.0	43	54.4	
Result	Negative	11	44.0	7	8.9	**< 0.001** ^*^
	Positive	14	56.0	72	91.1	

*χ*^2^ Chi square test

*p*: *p* value for comparing the two studied groups

^*^ Statistically significant at *p* ≤ 0.05

Seven cases of that showing a moderate SLP-2 intensity were interrupted as negative, hence they showed a proportion of staining of less than 10%

The bold is the statistically significant results

Moreover, samples of papillary thyroid carcinoma showed a mean proportion of positively stained cells for SLP-2 of 64.49 ± 32.59, this was statistically significantly higher than the mean proportion of 25.85 ± 33.11 of cells in other thyroid lesions group (*p* ˂0.001).

**Fig. 4:**
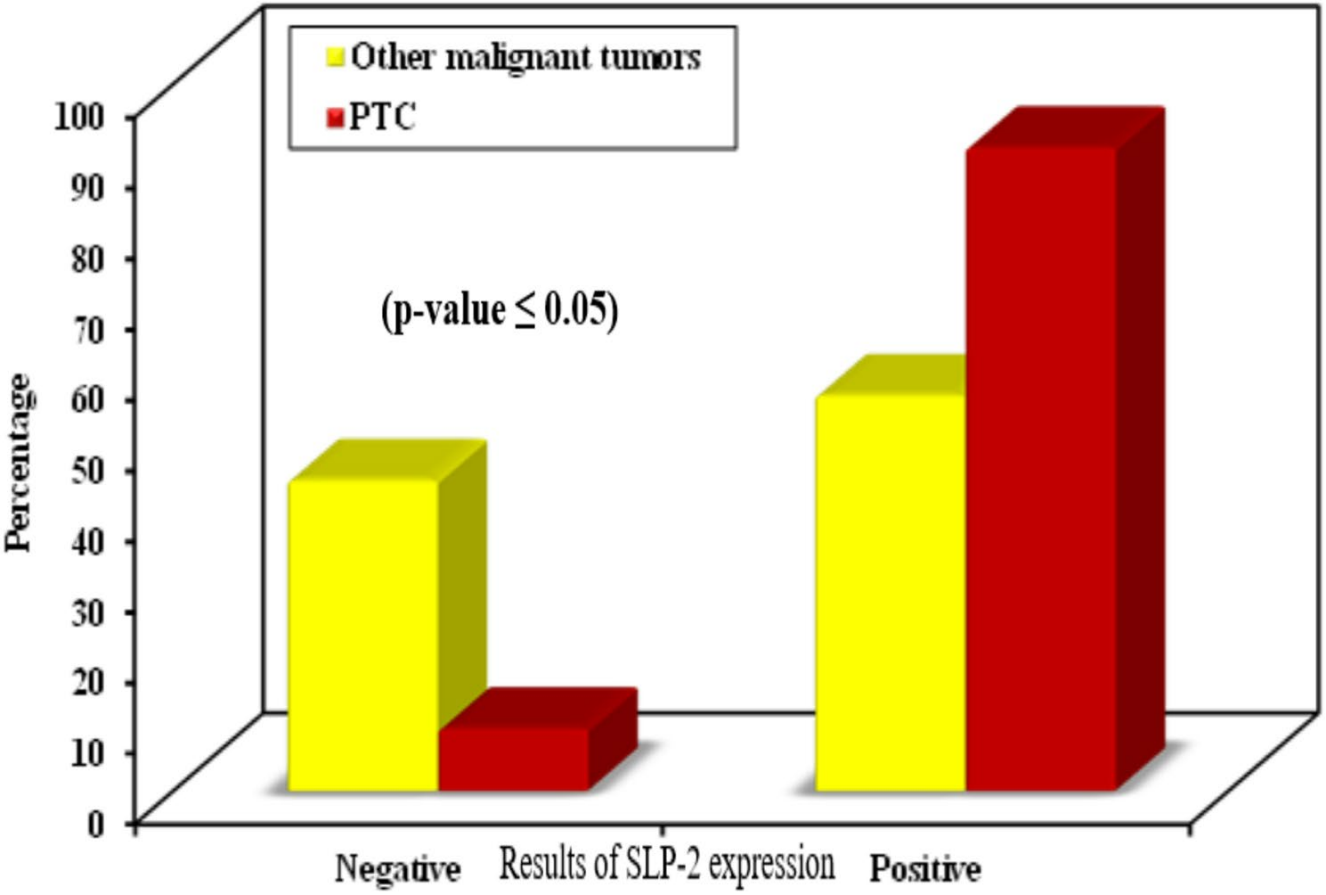
Distribution of cases of PTC and other malignant thyroid carcinoma cases according to results of SLP-2 expression

There are 25 cases of other malignant thyroid carcinomas, 14 samples of them have shown positive SLP-2 expression representing 56% of cases. Samples of papillary thyroid carcinoma have shown a statistically significant association with positive expression as well as stronger expression of SLP-2 in relation to other malignant thyroid carcinomas (*p* ˂0.001) (Fig. 4 and Table 4).

Moreover, cases of papillary thyroid carcinoma showed a statistically significant higher mean proportion of positively stained cells for SLP-2 of 64.49 ± 32.59, compared to 38.20 ± 36.85 of cells in other malignant thyroid carcinomas group (*p* = 0.002).

### TROP-2 immunohistochemical expression

**Fig. 5:**
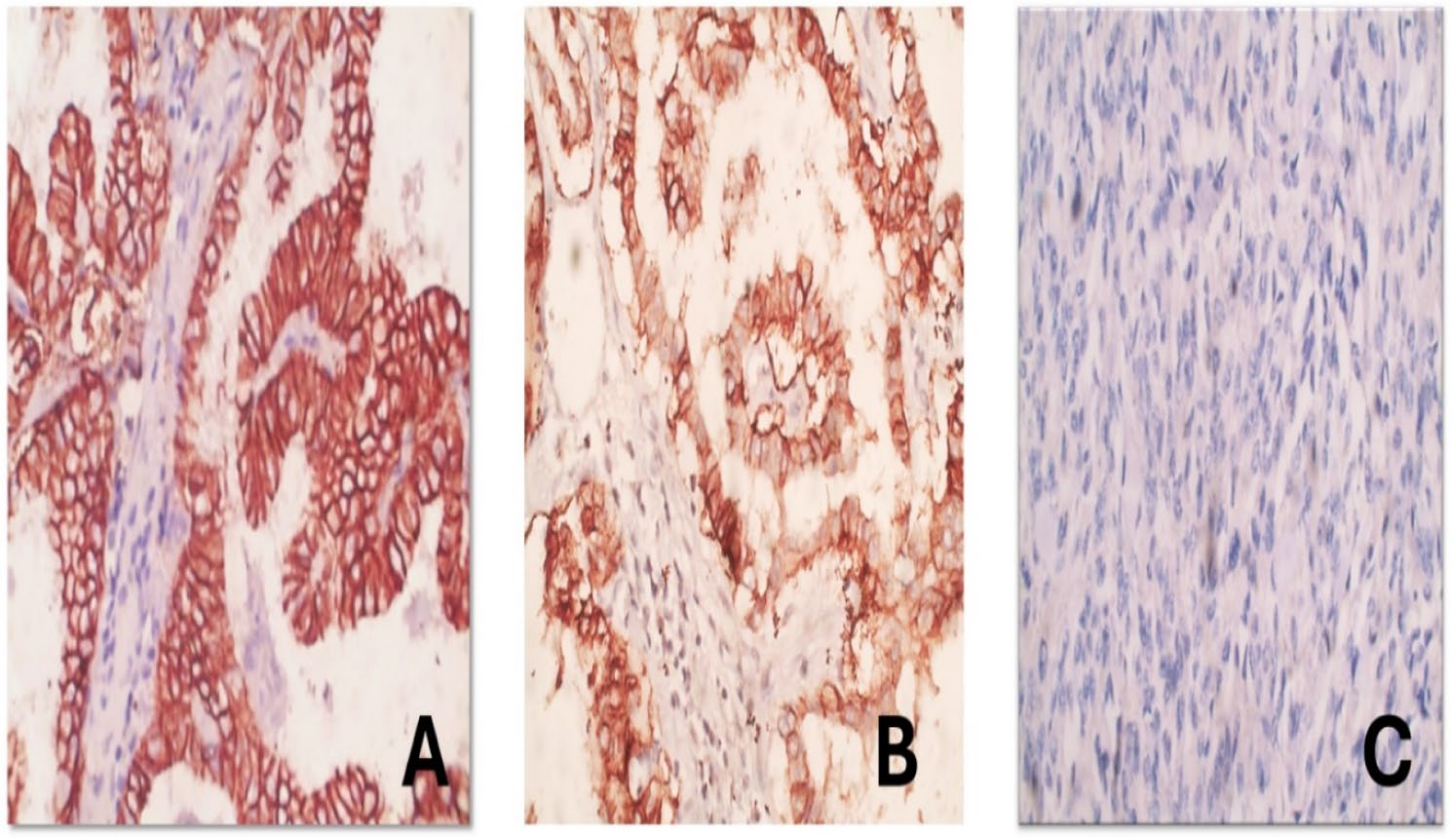
(**A**) Strong positive membranous immunohistochemical expression of TROP-2 in 100% of tumor cells in a case of papillary thyroid carcinoma (TROP-2, DAB, hematoxylin, 400x). (**B**) Moderately positive membranous immunohistochemical expression of TROP-2 in 80% of tumor cells in a case of papillary thyroid carcinoma (TROP-2, DAB, hematoxylin, 400x). (**C**) Negative immunohistochemical expression of TROP-2 in tumor cells in a case of anaplastic thyroid carcinoma (TROP-2, DAB, hematoxylin, 400x)

Cases were immunohistochemically stained for TROP-2, seventy cases were positive for TROP-2, representing 48.6% of all cases; 47 cases showed strong TROP-2 expression, 23 cases showed moderate TROP-2 expression, 12 cases showed faint non-conclusive expression of TROP-2, and 62 cases showed no TROP-2 expression (Fig. 5).

Sixty-five cases of PTC group out of 79 were positive for TROP-2, representing 82.3% of group cases. 5 cases of other thyroid lesions out of 65 were positive for TROP-2, representing 7.7% of group cases.

**Fig. 6:**
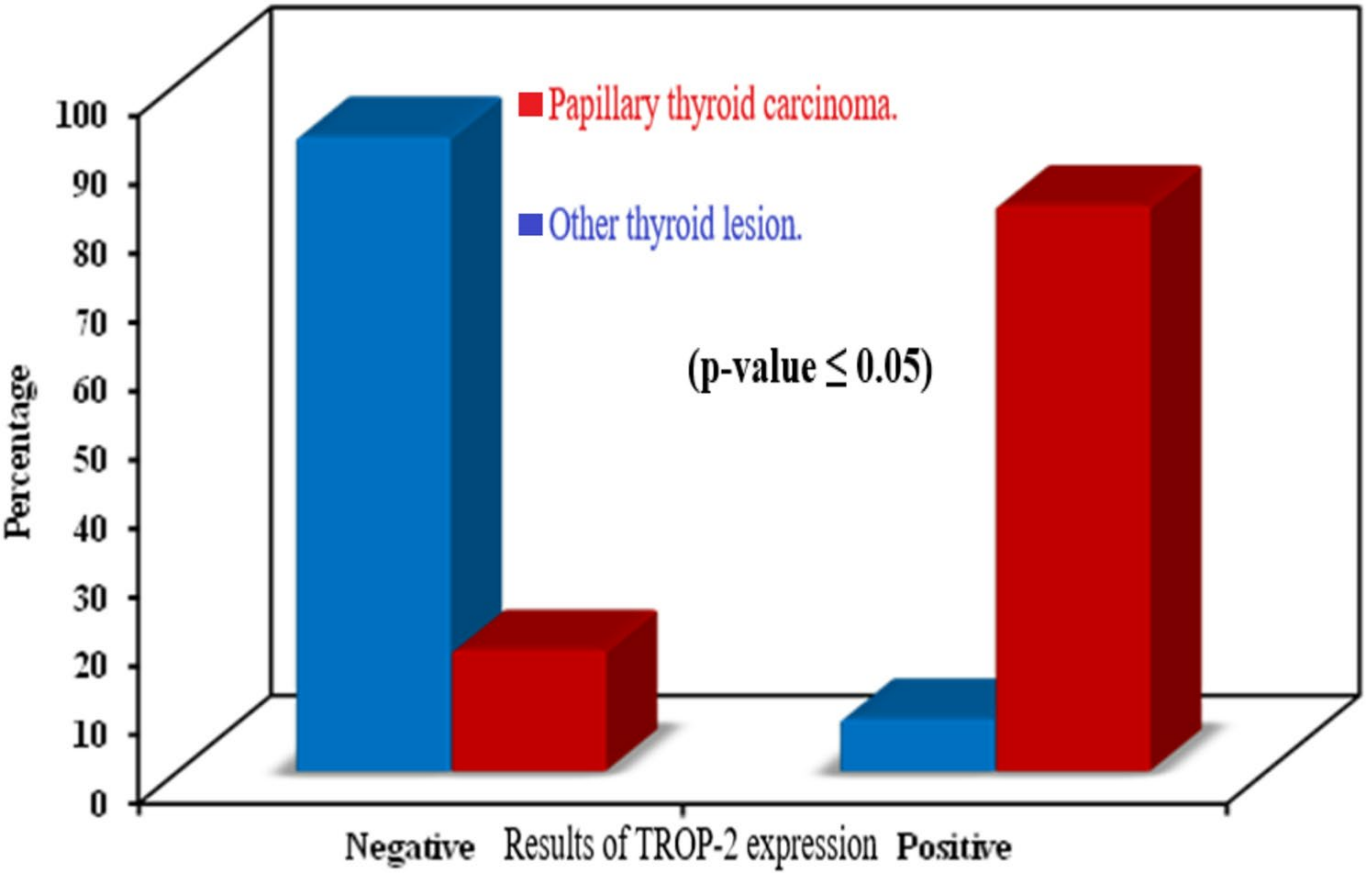
Distribution of cases of PTC and other thyroid lesions according to results of TROP-2 expression

Cases of papillary thyroid carcinoma have a statistically significant positive expression as well as stronger expression for TROP-2 in comparison with other thyroid lesions (*p* ˂0.001) (Table 5 and Fig. 6).

**Table 5:** Distribution of cases of PTC, other thyroid lesions and other malignant tumors according to results of TROP-2 expression (*n* = 144 & 104 respectively)

TROP-2 expression		Other thyroid lesions (*n* = 65)		PTC (*n* = 79)		*p*-value
		No.	%	No.	%	
Intensity	Negative	48	73.8	14	17.7	**< 0.001** ^*^
	Faint	12	18.5	0	0.0	
	Moderate	3	4.6	20	25.3	
	Strong	2	3.1	45	57.0	
Result	Negative	60	92.3	14	17.7	**< 0.001** ^*^
	Positive	5	7.7	65	82.3	

TROP-2 expression		**Other malignant tumors (** ***n*** ** = 25)**		**PTC** **(** ***n*** ** = 79)**		**p-value**
		**No.**	**%**	**No.**	**%**	
Intensity	Negative	13	52.0	14	17.7	**< 0.001** ^*^
	Faint	10	40.0	0	0.0	
	Moderate	1	4.0	20	25.3	
	Strong	1	4.0	45	57.0	
Result	Negative	23	92.0	14	17.7	**< 0.001** ^*^
	Positive	2	8.0	65	82.3	

*χ*^2^ Chi square test

*p*: *p* value for comparing the two studied groups

^*^ Statistically significant at *p* ≤ 0.05

The bold is the statistically significant results

Moreover, cases of papillary thyroid carcinoma showed a statistically significant higher mean proportion of positively stained cells for TROP-2 of 49.18 ± 37.56, compared to 6.38 ± 12.67 of cells in other thyroid lesions group (*p* ˂0.001).

**Fig. 7:**
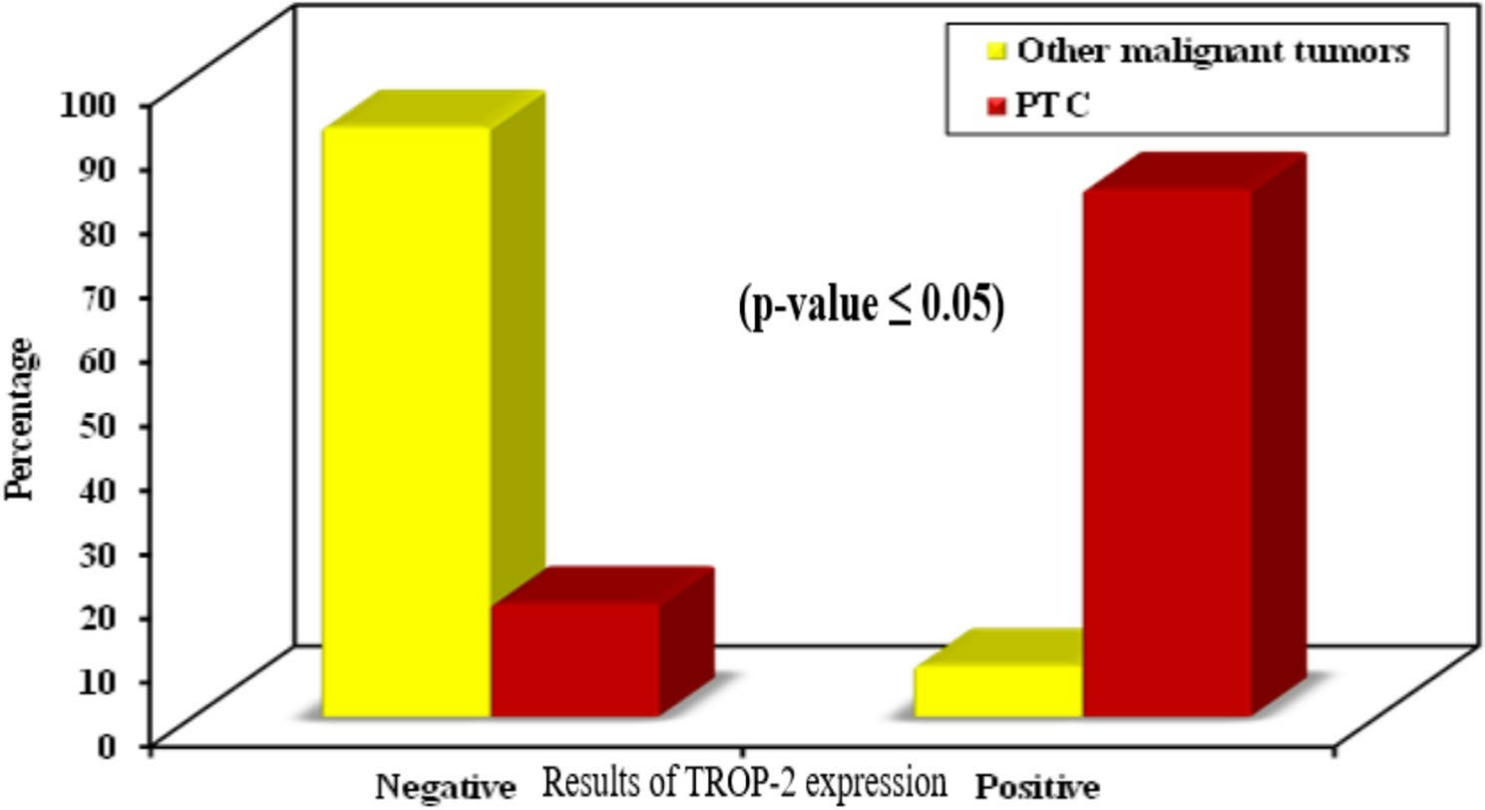
Distribution of cases of PTC and other malignant thyroid carcinomas according to results of TROP-2 expression

There are 25 cases of other malignant thyroid carcinomas, 2 of them showed positive TROP-2 expression representing 8% of cases. Cases of papillary thyroid carcinoma have a statistically significant positive expression for TROP-2 than other malignant thyroid carcinomas (*p* ˂0.001) (Table 5 and Fig. 7). Also, cases of papillary thyroid carcinoma have a statistically significant stronger expression of TROP-2 than other malignant thyroid carcinoma cases (*p* ˂0.001) (Table 5).

Moreover, cases of papillary thyroid carcinoma have a statistically significant higher mean proportion of positively stained cells for TROP-2 of 49.18 ± 37.56, compared to 9.40 ± 12.10 of cells in other malignant thyroid carcinomas group (*p* ˂0.001).

#### Diagnostic performance of SLP-2

This was measured to discriminate papillary thyroid carcinoma from other thyroid lesions. According to the receiver-operating characteristic (ROC) curve; the area under the curve is 0.791 for SLP-2 proportion of expression.

**Fig. 8:**
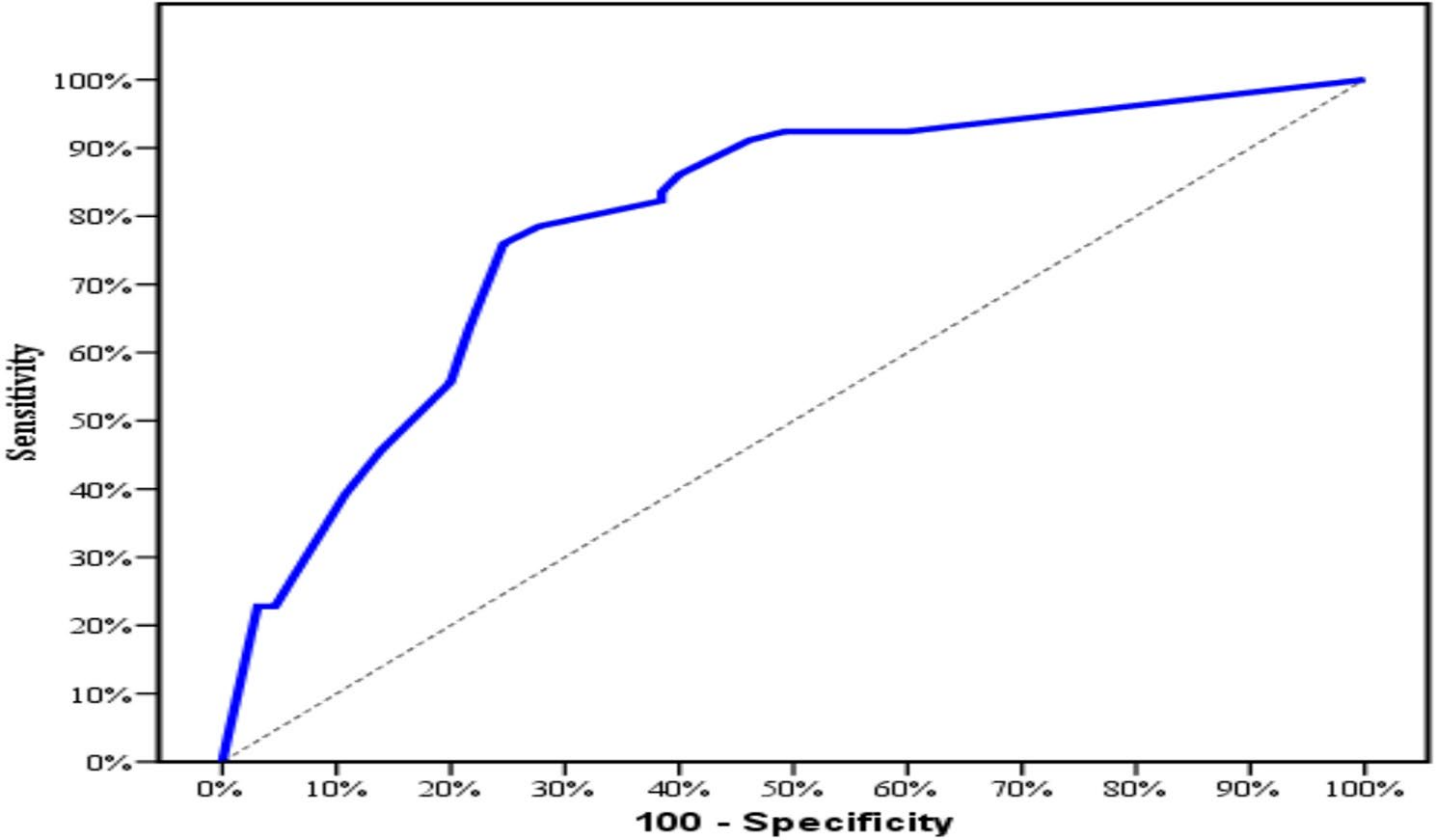
ROC curve for SLP-2 proportion to discriminate PTC patients (*n* = 79) from other thyroid lesions (*n* = 65)

The calculated sensitivity is 78.48% and the calculated specificity is 72.31%. The calculated positive predictive value is 77.5%, and the negative predictive value is 73.4%. Also, the calculated cut off point for the extent of positively stained cells for SLP-2 to discriminate papillary thyroid carcinoma from other thyroid lesions was estimated to be more than 30% of tumor cells (Table 6 and Fig. 8). This is the suggested cut off point for SLP2 proportion of staining to be used in future researches for better discrimination between PTC and other thyroid lesion.

**Table 6:** Diagnostic performance for SLP-2 and TROP-2 to discriminate PTC patients (*n* = 79) from other thyroid lesions (*n* = 65)

	Accuracy	*p*-value	Sensitivity	Specificity	PPV	NPV	Cut off point
SLP-2 expression	77.78	**< 0.001** ^*^	91.14	61.54	74.23	85.11	> 30
TROP-2 expression	0.844	**< 0.001** ^*^	81.01	83.08	85.3	78.3	> 10
Combined SLP-2 and TROP-2 expression	0.870	**< 0.001** ^*^	86.96	74.67	75.95	86.15	> 30 and > 10 respectively

*AUC* Area Under a Curve, *p* *value* Probability value, *NPV* Negative predictive value, *PPV* Positive predictive value

^*^ Statistically significant at *p* ≤ 0.05

^#^ Cut off was chosen according to Youden index

The bold is the statistically significant results

#### Diagnostic performance of TROP-2

This was measured to discriminate papillary thyroid carcinoma from other thyroid lesions. According to the receiver-operating characteristic (ROC) curve, the area under the curve is 0.844 for TROP-2 proportion of expression.

**Fig. 9:**
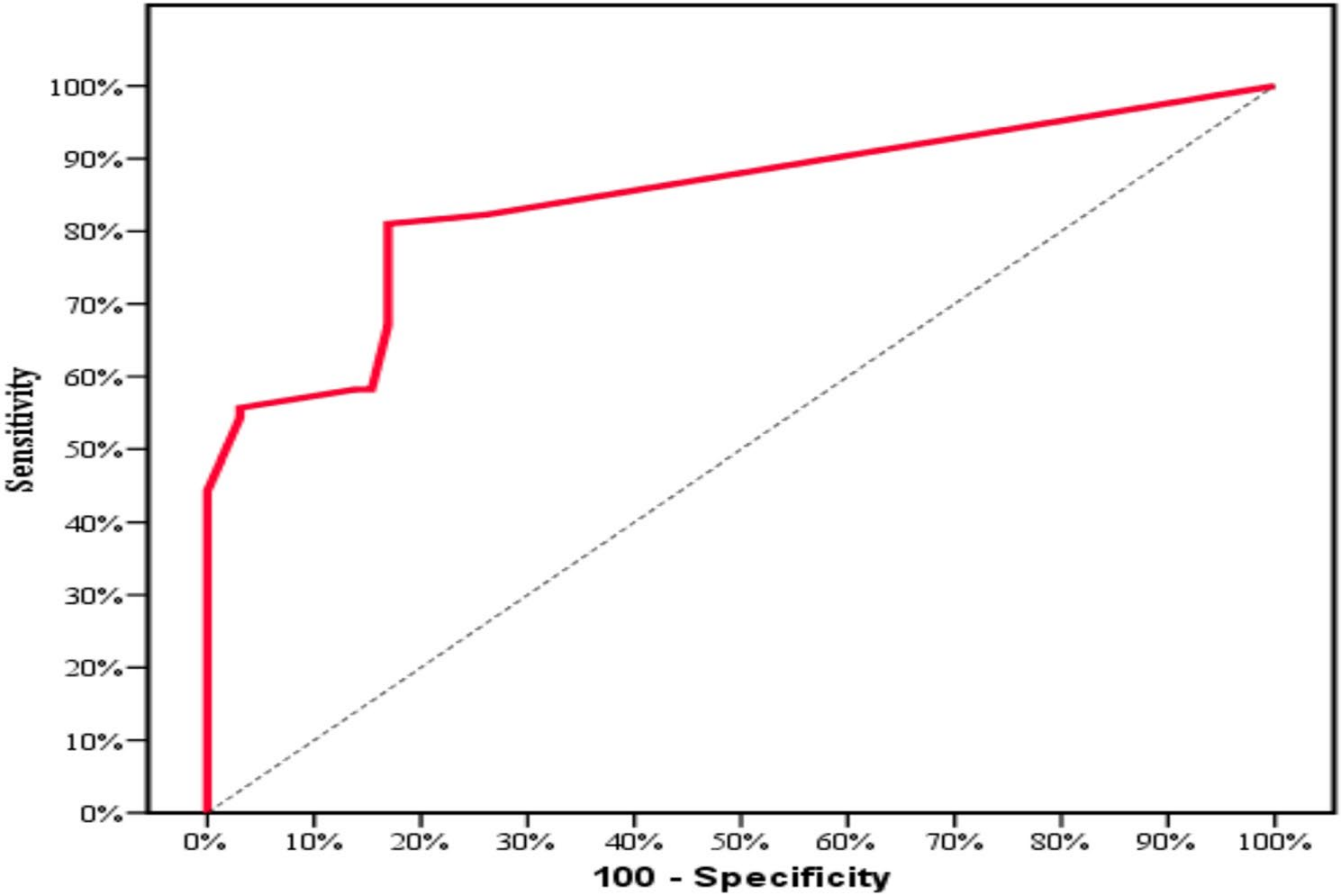
ROC curve for TROP-2 to discriminate PTC patients (*n* = 79) from other thyroid lesions (*n* = 65)

The calculated sensitivity is 81.01% and the calculated specificity is 83.08%. The calculated positive predictive value is 85.3%, and the negative predictive value is 78.3%. Also, the calculated cut off point for the extent of positively stained cells for TROP-2 to discriminate papillary thyroid carcinoma from other thyroid lesions was estimated to be more than 10% of tumor cells (Table 6 and Fig. 9).

#### Combined diagnostic performance of SLP-2 and TROP-2

**Fig. 10:**
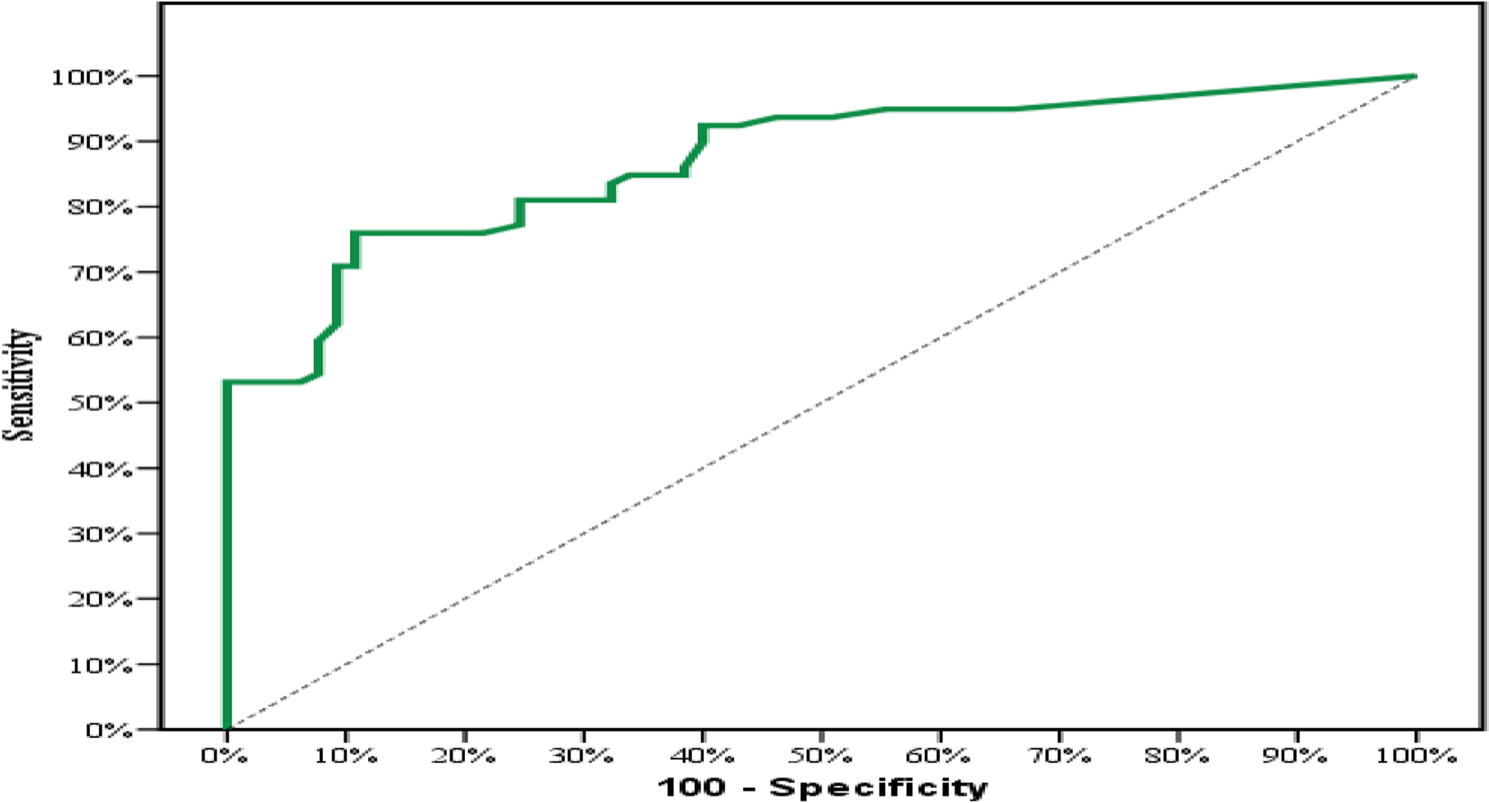
ROC curve for combined SLP-2 and TROP-2 proportion to discriminate PTC cases (*n* = 79) from other thyroid lesions (*n* = 65)

Based on the proportion of expression of both TROP-2 and SLP-2; combined diagnostic performance was assessed. This raised the test sensitivity to 86.96% and lowered the specificity to 74.67%. Also, it lowered the positive predictive value to 75.95%, yet it raised the negative predictive value to 86.15%. The area under the curve is 0.870 (Table 6 and Fig. 10).

#### Combined results of expression of SLP-2 and TROP-2

SLP-2 calculated sensitivity is 91.14% and the calculated specificity is 61.54%. The calculated positive predictive value is 74.23%, the negative predictive value is 85.11% and the calculated accuracy is 77.78% (Table 7). TROP-2 calculated sensitivity is 82.28% and the calculated specificity is 92.31%. The calculated positive predictive value is 92.86%, the negative predictive value is 81.08% and the test accuracy is 86.81 (Table 7).

**Table 7:** Cut off values of SLP-2 results, TROP-2 results and combined results of both for to discriminate PTC patients (*n* = 79) from other thyroid lesions (*n* = 65)

	Other thyroid lesions (*n* = 65)		PTC (*n* = 79)		Sensitivity	Specificity	PPV	NPV	Accuracy	*P*-value
	No.	%	No.	%						
SLP-2 results										
Negative	40	61.5	7	8.9	91.14	61.54	74.23	85.11	77.78	< 0.001*
Positive	25	38.5	72	91.1						
TROP-2 results										
Negative	60	92.3	14	17.7	82.28	92.31	92.86	81.08	86.81	< 0.001*
Positive	5	7.7	65	82.3						
Results of combined positivity of either SLP-2 or TROP-2										
Negative	36	55.4	5	6.3	93.67	55.38	71.84	87.80	76.39	< 0.001*
Positive	29	44.6	74	93.7						
Results of combined positivity of both SLP-2 and TROP-2										
Negative	64	98.5	16	20.3	79.75	98.46	98.44	80.0	88.19	< 0.001*
Positive	1	1.5	63	79.7						

*NPV* Negative predictive value, *PPV* Positive predictive value

^*^ Statistically significant at *p* ≤ 0.05

The bold is the statistically significant results

Combining results of SLP-2 and TROP-2 have been done by two different methods, first the cases were considered positive when they are positive for either SLP-2 or TROP-2. Cases of papillary thyroid carcinoma showed a statistically significant association with positive expression for either SLP-2 or TROP-2 more than other thyroid lesions (p-Value ˂0.0001), this raised the test sensitivity to 93.67% and lowered the specificity to 55.38%. Also, it lowered the positive predictive value to 71.84%, yet it raised the negative predictive value to 87.80%. The test accuracy is 76.39%. (Table 7).

Then the cases showing positive expressions for both SLP-2 and TROP-2 were considered positive. Papillary thyroid carcinoma cases have a statistically significant association with positive expression for both SLP-2 and TROP-2 in comparison with other thyroid lesions (*p* ˂0.0001). Moreover, all other malignant thyroid cases (25 cases) were negative for combined SLP-2 and TROP-2 expressions (Table 7).

This showed a slightly lower sensitivity of 79.75% and an extremely high specificity of 98.46%. Also, it showed an extremely high positive predictive value of 98.44%, yet it slightly lowered the negative predictive value to 80.0%. The test accuracy is highest in this method of interpretation, with estimated accuracy of 88.19% (Table 7).

#### Association between SLP-2 expression and variable histopathological and demographic characteristics

There is positive SLP-2 expression in 63.75% of female cases (74 out of 112) and in 71.88% of male cases (23 out of 32). Therefore, there is no statistically significant association between the expression of SLP-2 and the gender of cases (*p* = 0.54).

SLP-2 showed positive expression in 65.09% of cases less than 55 years old (69 out of 106) and in 68.42% of cases more than 55 years old (26 out of 38) with no statistically significant association between the expression of SLP-2 and the age of cases (*p* = 0.71).

The rest of histopathological characteristics are only limited to malignant cases (104 cases), such as lymphovascular invasion, perineural invasion, capsular invasion, extracapsular extension, tumor size, nodal metastasis and tumor stage.

There is positive SLP-2 expression in 82.1% of malignant thyroid female cases (64 out of 78) and in 84.6% of malignant thyroid male cases (22 out of 26) with no statistically significant association between the expression of SLP-2 and the gender of malignant cases (*p* = 0.76).

88% of malignant thyroid cases less than 55 years old were positive for SLP-2 (60 out of 68), while 72.2% of malignant cases 55 years old or more are positive for SLP-2 (26 out of 36), there is a statistically significant association between younger cases and positive SLP-2 expression compared to older cases (*p* = 0.04).

Malignant tumors with no perineural invasion have shown positive expression for SLP-2 in 86.2% (81 out of 94), compared to 50% of cases with evidence of perineural invasion have shown positive SLP-2 expression (5 out of 10), thus malignant thyroid cases with no evidence of perineural invasion have a statistically significant association with positive SLP-2 expression than cases with evidence of perineural invasion (*p* = 0.013) (Table 8).

**Table 8:** Distribution of results of SLP-2 expression in malignant thyroid cases according to variable histopathological parameters (*n* = 104)

		N	SLP-2 Result				*p*-value
			Negative (*n* = 18)		Positive (*n* = 86)		
			No.	%	No.	%	
Perineural invasion state	No	94	13	13.8	81	86.2	^**FE**^ **p=** **0.013** ^*^
	Yes	10	5	50.0	5	50.0	
Lymphovascular invasion state	No	72	9	12.5	63	87.5	0.052
	Yes	32	9	28.1	23	71.9	
Capsule invasion	No	69	10	14.5	59	85.5	0.287
	Yes	35	8	22.9	27	77.1	
Extra-thyroid extension	No	77	13	16.9	64	83.1	^FE^p= 1.000
	Yes	27	5	18.5	22	81.5	
T stage	T1a	14	1	7.1	13	92.9	0.423
	T1b	12	3	25.0	9	75.0	
	T2	16	2	12.5	14	87.5	
	T3a	29	7	24.1	22	75.9	
	T3b	31	4	12.9	27	87.1	
	T4a	2	1	50.0	1	50.0	
Lymph nodal metastasis stage	NX/0	82	15	18.3	67	81.7	^FE^p= 0.758
	N1	22	3	13.6	19	86.4	
Stage	I	73	9	12.3	64	87.7	**0.003** ^*^
	II	24	4	16.7	20	83.3	
	III	2	1	50.0	1	50.0	
	IV	5	4	80.0	1	20.0	

*χ*^*2*^ Chi square test, *FET* Fisher Exact test

*p*: *p* value for relation SLP2 result and different parameters

﻿The bold is the statistically significant results

Malignant tumors with no evidence of lymphovascular invasion have shown positive expression for SLP-2 in 87.5% (63 out of 72), while 71.9% of cases with evidence of lymphovascular invasion have shown positive SLP-2 expression (23 out of 32). Yet, the difference is insignificant despite a low *p*-value (0.052) (Table 8).

There is a positive SLP-2 expression in 77.1% (27 out of 35) of malignant thyroid cases with thyroid capsular invasion and in 85.5% of cases (59 out of 69) with no detected thyroid capsular invasion. The difference is insignificant (Table 8).

SLP-2 expression has been considered positive in 81.5% of malignant cases with extra-thyroidal extension (22 out of 27) and in 83.1% of cases with no detected extrathyroidal extension (64 out of 77). Also, The difference between the two groups is insignificant (Table 8).

Regarding T stage, SLP-2 has been positive in 92.9%, 75.0%, 87.5%, 75.9%, 87.1% and 50.0% of T1a, T1b, T2, T3a, T3b and T4 respectively. There is no statistically significant association between the expression of SLP-2 and T stage (Table 8).

Regarding the status of lymph nodal metastasis, SLP-2 has been positive in 81.7% of cases with no detected lymph nodal invasion (Nx or N0) (67 out of 82) and in 86.4% cases showing evidence of regional nodal metastasis (19 out of 22). There is no statistically significant association between the expression of SLP-2 and status of lymph nodal metastasis (Table 8).

Regarding the stage of malignant thyroid cases, SLP-2 has been positive in 87.7% (64 out of 73), 83.3% (20 out of 24), 50.0% (1 out of 2) and 20.0% (1 out of 5) of stage I, II, III and IV respectively. The difference between stages of malignant cases is statistically significant (*p* = 0.003) (Table 8).

Regarding the results of SLP-2 expression in papillary thyroid carcinoma group, there was no statistically significant association between SLP-2 expression results and variable demographic and histopathological characteristics (Table 9).

**Table 9:** Distribution of results of SLP-2 expression according to variable demographic and histopathological parameters in PTC cases (*n* = 79)

		N	SLP-2 Result				*p*
			Negative (*n* = 7)		Positive (*n* = 72)		
			No.	%	No.	%	
Gender	Male	21	2	9.5	19	90.5	^FE^p= 1.000
	Female	58	5	8.6	53	91.4	
Age	Less than 55	64	6	9.4	58	90.6	^FE^p= 1.000
	55 or more	15	1	6.7	14	93.3	
Perineural invasion state	No	74	6	8.1	68	91.9	^FE^p= 0.379
	Yes	5	1	20.0	4	80.0	
Lymphovascular invasion State	No	61	6	9.8	55	90.2	^FE^p= 1.000
	Yes	18	1	5.6	17	94.4	
Thyroid capsular invasion	No	55	6	10.9	49	89.1	^FE^p= 0.669
	Yes	24	1	4.2	23	95.8	
Extra thyroid extension	No	57	6	10.5	51	89.5	^FE^p= 0.666
	Yes	22	1	4.5	21	95.5	
T stage	T1a	14	1	7.1	13	92.9	0.186
	T1b	10	1	10.0	9	90.0	
	T2	15	2	13.3	13	86.7	
	T3a	18	2	11.1	16	88.9	
	T3b	20	0	0.0	20	100.0	
	T4a	2	1	50.0	1	50.0	
LN stage	NX/0	61	7	11.5	54	88.5	^FE^p= 0.341
	N1	18	0	0.0	18	100.0	
Stage	I	68	6	8.8	62	91.2	0.196
	II	9	0	0.0	9	100.0	
	III	2	1	50.0	1	50.0	
	IV	0	0	0.0	0	0.0	

*χ*^*2*^ Chi square test, *FET* Fisher Exact test

*p*: *p* value for relation SLP2 result and different parameters

Regarding the mean proportion of positive SLP-2 cells expression in the papillary thyroid carcinoma group, there was a statistically significant association between the mean proportion of stained tumor cells and the age of cases (*p* = 0.01). The mean of stained tumor cells in cases 55 years or older is 83.33 ± 25.82, while the mean of stained tumor cells in cases less than 55 years old is 60.08 ± 32.59.

The rest of demographic and histopathological characteristics have no statistically significant association with SLP-2 proportion of expression in papillary thyroid carcinoma group.

Regarding the intensity of SLP-2 expression in the papillary thyroid carcinoma group, there is a statistically significant association between the intensity of stained tumor cells and cases younger than 55 years old (*p* = 0.008) (Table 10). Also, there is a statistically significant association between the intensity of SLP-2 stained tumor cells and the stage of cases of PTC (*p* = 0.040). Cases of stage II compared with variable stages have a statistically significant association with stronger SLP2 expression.

**Table 10:** Distribution of SLP-2 result with demographic and histopathological data and in other malignant thyroid tumors group (*n* = 25)

		N	SLP-2 Result				*p*
			Negative (*n* = 11)		Positive (*n* = 14)		
			No.	%	No.	%	
Gender	**Male**	5	2	40.0	3	60.0	^FE^p= 1.000
	**Female**	20	9	45.0	11	55.0	
Age	**Less than 55**	4	2	50.0	2	50.0	^FE^p= 1.000
	**55 or more**	21	9	42.9	12	57.1	
PN state	**No**	20	7	35.0	13	65.0	^FE^p= 0.133
	**Yes**	5	4	80.0	1	20.0	
LV State	**No**	11	3	27.3	8	72.7	^FE^p= 0.227
	**Yes**	14	8	57.1	6	42.9	
Capsule Invasion	**No**	14	4	28.6	10	71.4	^FE^p= 0.116
	**Yes**	11	7	63.6	4	36.4	
Extra thyroid extension	**No**	20	7	35.0	13	65.0	^FE^p= 0.133
	**Yes**	5	4	80.0	1	20.0	
T stage	**T1a**	0	0.0	0	0.0	0.0	0.487
	**T1b**	2	2	100.0	0	0.0	
	**T2**	1	0	0.0	1	100.0	
	**T3a**	11	5	45.5	6	54.5	
	**T3b**	11	4	36.4	7	63.6	
	**T4a**	0	0	0.0	0	0.0	
LN stage	**NX/0**	21	8	38.1	13	61.9	^FE^p= 0.288
	**N1**	4	3	75.0	1	25.0	
Stage	**I**	5	3	60.0	2	40.0	0.123
	**II**	15	4	26.7	11	73.3	
	**III**	0	0	0.0	0	0.0	
	**IV**	5	4	80.0	1	20.0	

*χ*^*2*^ Chi square test, *FET* Fisher Exact test

*p*: *p* value for Relation SLP2 Result and different parameters

﻿The bold is the statistically significant results

The rest of demographic and histopathological characteristics have no statistically significant association with SLP-2 proportion of expression in papillary thyroid carcinoma group.

Regarding the group of other thyroid lesions, there is no statistically significant association between SLP-2 expression results and variable demographic and histopathological parameters (Table 10).

#### Association between TROP-2 expression and variable histopathological and demographic characteristics

There is positive TROP-2 expression in 50% of female cases (56 out of 112) and in 43.75% of male cases (14 out of 32). The difference is statistically insignificant (*p* = 0.53). TROP-2 showed a positive expression in 51.89% of cases less than 55 years old (55 out of 106) and in 39.47% of cases more than 55 years old (15 out of 38). The difference is statistically insignificant (*p* = 0.19).

The rest of histopathological characteristics are only limited to malignant cases (104 cases), such as lymphovascular invasion, perineural invasion, capsular invasion, extra-capsular extension, tumor size, nodal metastasis and tumor stage.

Malignant cases with no perineural invasion have shown positive expression for TROP-2 in 67% (63 out of 94), while 40% of cases with evidence of perineural invasion have shown positive TROP-2 expression (4 out of 10). There is no statistically significant association between the expression of TROP-2 and the status of perineural invasion (Table 11).

**Table 11:** Distribution of results of TROP-2 expression in malignant thyroid cases according to variable histopathological parameters (*n* = 104)

		N	TROP-2 Result				*p*-value
			Negative (*n* = 37)		Positive (*n* = 67)		
			No.	%	No.	%	
Perineural invasion state	No	**94**	31	33.0	63	67.0	^FE^p= 0.161
	Yes	**10**	6	60.0	4	40.0	
Lymphovascular invasion state	No	**72**	19	26.4	53	73.6	**0.003** ^*^
	Yes	**32**	18	56.3	14	43.8	
Capsular invasion	No	**69**	21	30.4	48	69.6	^FE^p= 0.124
	Yes	**35**	16	45.7	19	54.3	
Extra thyroid extension	No	**77**	27	35.1	50	64.9	0.854
	Yes	**27**	10	37.0	17	63.0	
T stage	T1a	**14**	3	21.4	11	78.6	0.401
	T1b	**12**	3	25.0	9	75.0	
	T2	**16**	4	25.0	12	75.0	
	T3a	**29**	11	37.9	18	62.1	
	T3b	**31**	15	48.4	16	51.6	
	T4a	**2**	1	50.0	1	50.0	
LN stage	NX/0	**82**	29	35.4	53	64.6	0.931
	N1	**22**	8	36.4	14	63.6	
Stage	I	**73**	17	23.3	56	76.7	**< 0.001** ^*^
	II	**24**	14	58.3	10	41.7	
	III	**2**	1	50.0	1	50.0	
	IV	**5**	5	100.0	0	0.0	

*χ*^*2*^ Chi square test, *FET* Fisher Exact test

*p*: *p* value for Relation TROP2 result and different parameters

^*^ Statistically significant at *p* ≤ 0.05

The bold is the statistically significant results

Samples showing no evidence of lymphovascular invasion have a positive expression for TROP-2 in 73.6% (53 out of 72). Also, 43.8% of cases with evidence of lymphovascular invasion have shown positive TROP-2 expression (14 out of 32). Thus, malignant cases with no evidence of lymphovascular invasion have a statistically significant higher association with positive TROP-2 expression (*p* = 0.003) (Table 11).

TROP-2 is positive in 54.3% (19 out of 35) of malignant thyroid cases showing thyroid capsular invasion and in 69.6% of cases (48 out of 69) with no detected thyroid capsular invasion. There is no statistically significant association between the expression of TROP-2 and thyroid capsular invasion in malignant thyroid cases (Table 11).

TROP-2 expression has been considered positive in 63% of malignant cases with extra-thyroidal extension (17 out of 27) and in 64.9% of cases with no extrathyroidal extension detected (50 out of 77). Also, there is no statistically significant association between the expression of TROP-2 and extrathyroidal extension in malignant thyroid cases (Table 11).

Regarding T stage, TROP-2 has been positive in 78.6%, 75%, 75%, 62.1%, 51.6% and 50.0% of T1a, T1b, T2, T3a, T3b and T4 cases respectively. There is no statistically significant association between the expression of TROP-2 and tumor T stage in malignant thyroid cases (Table 11).

Regarding the status of lymph nodal metastasis, TROP-2 has been positive in 64.6% of cases with no detected lymph nodal invasion (Nx or N0) (53 out of 82) and in 63.6% cases showing evidence of regional nodal metastasis (14 out of 22). There is no statistically significant association between the expression of TROP-2 and status of lymph nodal metastasis in malignant thyroid cases (Table 11).

Regarding the stage of malignant thyroid cases, TROP-2 has been positive in 76.7% (56 out of 73), 41.7% (10 out of 24), 50.0% (1 out of 2) and 0% (0 out of 5) of stage I, II, III and IV cases respectively. The difference is statistically significant (*p* ˂0.001) (Table 11).

Female patients of PTC show a statistically significant association with positive TROP-2 expression in comparison with male PTC samples. There is positive TROP-2 expression in 87.9% (51 out of 58) of female samples and 66.7% (14 out of 21) of male samples (*p*˂ 0.05). On the other hand, the rest of other variable demographic and histopathological parameters have no statistically significant association with TROP-2 expression results in PTC group **(**Table 12).

**Table 12:** Distribution of results of TROP-2 expression according to variable demographic and histopathological parameters in PTC cases (*n* = 79)

		N	TROP-2 Result				*p*-value
			Negative (*n* = 14)		Positive (*n* = 65)		
			No.	%	No.	%	
Gender	**Male**	**21**	**7**	**33.3**	**14**	**66.7**	^**FE**^ **p=** 0.044*
	**Female**	**58**	**7**	**12.1**	**51**	**87.9**	
Age	**Less than 55**	**64**	**13**	**20.3**	**51**	**79.7**	^**FE**^ **p=** **0.285**
	**55 or more**	**15**	**1**	**6.7**	**14**	**93.3**	
Perineural invasion state	**No**	**74**	**13**	**17.6**	**61**	**82.4**	^**FE**^ **p=** **1.000**
	**Yes**	**5**	**1**	**20.0**	**4**	**80.0**	
Lymphovascular invasion State	**No**	**61**	**9**	**14.8**	**52**	**85.2**	^**FE**^ **p=** **0.290**
	**Yes**	**18**	**5**	**27.8**	**13**	**72.2**	
Capsule Invasion	**No**	**55**	**9**	**16.4**	**46**	**83.6**	^**FE**^ **p=** **0.750**
	**Yes**	**24**	**5**	**20.8**	**19**	**79.2**	
Extra thyroid extension	**No**	**57**	**9**	**15.8**	**48**	**84.2**	^**FE**^ **p=** **0.518**
	**Yes**	**22**	**5**	**22.7**	**17**	**77.3**	
T stage	**T1a**	**14**	**3**	**21.4**	**11**	**78.6**	**0.710**
	**T1b**	**10**	**1**	**10.0**	**9**	**90.0**	
	**T2**	**15**	**3**	**20.0**	**12**	**80.0**	
	**T3a**	**18**	**2**	**11.1**	**16**	**88.9**	
	**T3b**	**20**	**4**	**20.0**	**16**	**80.0**	
	**T4a**	**2**	**1**	**50.0**	**1**	**50.0**	
LN stage	**NX/0**	**61**	**10**	**16.4**	**51**	**83.6**	^**FE**^ **p=** **0.726**
	**N1**	**18**	**4**	**22.2**	**14**	**77.8**	
Stage	**I**	**68**	**13**	**19.1**	**55**	**80.9**	**0.166**
	**II**	**9**	**0**	**0.0**	**9**	**100.0**	
	**III**	**2**	**1**	**50.0**	**1**	**50.0**	
	**IV**	**0**	**0**	**0.0**	**0**	**0.0**	

*χ*^*2*^ Chi square test, *FET* Fisher Exact test

*p*: *p *value for Relation TROP2 result and different parameters

^*^ Statistically significant at *p* ≤ 0.05

The bold is the statistically significant results

Regarding theitive SLP-2 expression in 63.75% of female cases ( proportion of TROP-2 expression in papillary thyroid carcinoma group, the mean of stained tumor cells in cases 55 years or older is 68.33 ± 37.64, while the mean of stained tumor cells in cases less than 55 years old is 44.69 ± 36.38. There is a statistically significant association between the mean proportion of stained tumor cells and the older age of patients (*p* = 0.02).

Also, in the papillary thyroid carcinoma group there is a statistically significant association between the proportion of TROP-2 stained tumor cells and lower stage (*p* = 0.02). The mean of stained tumor cells in stage II cases is 81.11 ± 19.65, while the mean of stained tumor cells in stage III cases is 35 ± 49.5.

However, the rest of other demographic and histopathological characteristics have no statistically significant association with TROP-2 proportion of expression in papillary thyroid carcinoma group.

Regarding the intensity of TROP-2 expression in the PTC group, there was no statistically significant association between the intensity of stained cells and demographic and histopathological characteristics.

Regarding the group of other thyroid lesions, there is no statistically significant association between TROP-2 expression results and variable demographic and histopathological parameters (Table 13).

**Table 13:** Distribution of TROP-2 result with demographic and histopathological data and in other malignant thyroid tumors group (*n* = 25)

		N	TROP-2 Result				*p*
			Negative (*n* = 23)		Positive (*n* = 2)		
			No.	%	No.	%	
Gender	**Male**	5	5	100.0	0	0.0	^FE^p= 1.000
	**Female**	20	18	90.0	2	10.0	
Age	**Less than 55**	4	3	75.0	1	25.0	^FE^p= 0.300
	**55 or more**	21	20	95.2	1	4.8	
PN state	**No**	20	18	90.0	2	10.0	^FE^p= 1.000
	**Yes**	5	5	100.0	0	0.0	
LV State	**No**	11	10	90.9	1	9.1	^FE^p= 1.000
	**Yes**	14	13	92.9	1	7.1	
Capsule Invasion	**No**	14	12	85.7	2	14.3	^FE^p= 0.487
	**Yes**	11	11	100.0	0	0.0	
Extra th. ex.	**No**	20	18	90.0	2	10.0	^FE^p= 1.000
	**Yes**	5	5	100.0	0	0.0	
T stage	**T1a**	0	0	0.0	0	0.0	0.597
	**T1b**	2	2	100.0	0	0.0	
	**T2**	1	1	100.0	0	0.0	
	**T3a**	11	9	81.8	2	18.2	
	**T3b**	11	11	100.0	0	0.0	
	**T4a**	0	0	0.0	0	0.0	
LN stage	**NX/0**	21	19	90.5	2	9.5	^FE^p= 1.000
	**N1**	4	4	100.0	0	0.0	
Stage	**I**	5	4	80.0	1	20.0	0.650
	**II**	15	14	93.3	1	6.7	
	**III**	0	0	0.0	0	0.0	
	**IV**	5	5	100.0	0	0.0	

*χ*^*2*^ Chi square test, *FET* Fisher Exact test

*p*: *p* value for relation SLP2 result and different parameters

The bold is the statistically significant results

Nineteen cases out of 20 of follicular variant of PTC cases have shown a positive expression for SLP-2 (95%) and 53 cases out of 59 of conventional papillary thyroid carcinoma group have shown positive expression for SLP-2 (89.83%), yet there is no statistically significant association between SLP-2 expression and variants of PTC in this study (Table 14).

**Table 14:** The relation between SLP-2 and TROP-2 expression results and variants of PTC (n=79)

SLP-2 expression		Conventional variant of PTC (*n* = 59)		Follicular variant of papillary thyroid carcinoma (*n* = 20)		*p*-value
		No.	%	No.	%	
Result	Positive	53	89.83	19	95	**0.6719** ^**fE**^
	Negative	6	10.17	1	5	

TROP-2 expression		**Conventional variant of PTC (** ***n*** ** = 59)**		**Follicular variant of papillary thyroid carcinoma** **(** ***n*** ** = 20)**		***p*** **-value**
		**No.**	**%**	**No.**	**%**	
Result	Positive	49	83.05	16	80	**0.7436** ^**fE**^
	Negative	10	16.95	4	20	

*FET* Fisher Exact test

The bold is the statistically significant results

Also, sixteen cases out of 20 of follicular variant of PTC cases have shown a positive expression for TROP-2 (80%) and 53 cases out of 59 of conventional papillary thyroid carcinoma group have shown positive expression for TROP-2 (83.05%), yet there is no statistically significant association between TROP-2 expression and variants of PTC in this study (Table 14).

## Discussion

Thyroid carcinoma is a common tumor affecting Egyptian population and internationally. This study has been conducted to assess SLP-2 and TROP-2 expression in papillary thyroid carcinoma and other thyroid lesions. Also, we have assessed their expression with demographic and histopathological parameters such as age at time of excision, gender, perineural invasion, lymphovascular invasion, thyroid capsular invasion, extra-thyroidal extension, tumor size, lymph node metastasis and tumor stage.

SLP-2 is an important mitochondrial membrane protein. SLP-2 protein has a role in regulation of proliferation, migration and invasion of many tumor cell types [5, 19]. It shows cytoplasmic or membranous staining in thyroid lesions [17].

TROP-2 is a transmembrane glycoprotein. Studies proved that it is also over-expressed in many carcinomas compared to normal tissues [9, 10, 20]. TROP-2 promotes tumor cellular migration and metastasis [21]. Also, it shows cytoplasmic or membranous staining in thyroid lesions [17].

This study has been conducted upon 144 cases of thyroid lesions, 79 of them of PTC and 65 of other thyroid lesions, including 19 cases of non-neoplastic cases, 21 benign cases and 25 cases of other malignant tumors, including 9 cases of follicular thyroid carcinomas, 11 cases of medullary thyroid carcinomas and five cases of anaplastic thyroid carcinomas.

In this study mouse monoclonal IgG SLP-2 antibody showed exclusive cytoplasmic staining with no nuclear or membranous staining. This pattern of expression of SLP-2 is similar to other studies by [17, 22–24], that all of them showed exclusive cytoplasmic staining of SLP-2 [17, 22, 23].

Yet, in Liu et al. study cases of PTC showed cytoplasmic staining with few cases showing concomitant membranous staining, this is due to a different clone of primary antibody, being a rabbit polyclonal antibody [24].

Seventy-two cases out of 79 (91.1%) of PTC were positive for SLP-2. On the other hand, 25 cases of other thyroid lesions out of 65 (38.5%) were positive for SLP-2. The difference is statistically significant (*p* ˂0.001). Similar statistically significant association was found in other studies with different levels of expression [17, 22–24].

In a study conducted by Yang et al. over 59 surgical specimens, it showed a relatively close result as 83.1% of cases of PTC and 20.7% of other thyroid lesions were considered SLP-2 positive with a *p*-value ˂0.05 [17]. In the study of Liu et al., over 107 cases of PTC with a control of surrounding normal thyroid tissue SLP-2 showed a statistically significant association with PTC carcinoma cases in relation to surrounding normal thyroid tissue with a *p*-value ˂0.001 [24].

In the Bartolome et al. study conducted upon 210 cases of benign and malignant thyroid lesions, 65% of PTC cases were positive for SLP-2, all cases of follicular adenoma were negative for SLP-2 and only a single case of follicular carcinoma and a single case of anaplastic carcinoma were positive for SLP-2. This variation in levels of expression is due to different producers of primary antibodies and different methods of evaluation as the study cut off point for positivity was more than 40% which is much higher than our study, being more than 10%, this explains the low levels of sensitivity and higher specificity. This is also in line with calculated data upon the test accuracy of SLP-2 in our study that suggested adoption of a cutoff point of 30% for better discrimination of both groups [22].

In the study performed by Attia et al. upon 75 cases of PTC cases and other thyroid lesions, 88.6% of PTC cases, which is close to our study, yet only 7.5% of other thyroid lesions were positive for SLP-2 (p-value is ˂ 0.001). The difference in percentage of expression is likely due to different sample sizes [23].

In our study, the calculated sensitivity is 91.14% and the calculated specificity is 61.54%. The calculated positive predictive value is 74.23%, the negative predictive value is 85.11% and the calculated accuracy is 77.78%. This was quite close to the study conducted by Yang that showed SLP-2 sensitivity of 83.3%, specificity of 79.3%, positive predictive value of 80.6, negative predictive value of 82.1% and diagnostic accuracy of 81.4% [17].

In this study there was no statistically significant association between the gender or age of cases in the PTC group and the expression of SLP-2. Also, this study concluded that there is no significant association between the gender or age of cases of other thyroid lesions and the expression of SLP-2.

Similar results were concluded by studies performed by Liu et al., Yang et al., Bartolome et al. and Attia et al. showing no statistically significant association between the gender or age of cases of PTC and SLP-2 expression [17, 22–24].

Also, there was no statistically significant association between all histopathological parameters such as perineural invasion, lymphovascular invasion, thyroid capsular invasion, extra-thyroidal extension, T-stage, lymph nodal metastasis and tumor stage and the expression of SLP-2 either in PTC group or other thyroid lesions group.

There is a statistically significant association between the mean proportion of SLP-2 expression and older age cases (*p* = 0.01). Also, there is statistically significant association between the stronger intensity of SLP-2 expression, older cases and stage II cases (*P* = 0.008 & 0.04 respectively).

Similar results were concluded by studies performed by Yang et al. and Attia et al. showing no statistically significant association between all histopathological parameters and expression of SLP-2 in PTC cases [17, 23]. Since, the two researchers have adopted interpretation criteria similar to our research.

On the other hand, the results of Liu et al. and Bartolome et al. stated that there is a statistically significant association between larger tumor size, presence of lymph nodal metastasis and higher stage in PTC cases and SLP-2 expression, also Bartolome showed a statistically significant association between presence of extra-thyroidal extension in PTC cases and SLP-2 expression, most likely due to different primary antibody provider and different method of interpretation of antibody positivity [17, 22, 24].

In our study mouse monoclonal IgG TROP-2 antibody showed membranous staining with few cases showing also cytoplasmic staining, no cases showing nuclear staining. This pattern of expression of TROP-2 is similar to the previous studies [17, 20, 25].

Yet, there are other studies that showed only membranous staining, likely due to different manufacturers of primary antibody, different clones and different antigen retrieval methods [23, 26–30].

Sixty-five cases out of 79 (82.3%) of PTC were positive for TROP-2, compared with 5 cases of other thyroid lesions out of 65 (7.7%) were positive for TROP-2. The difference is statistically significant (*p*-Value ˂0.001). Similar statistically significant association was found in other studies [17, 20, 23, 25, 29–31].

In a study conducted by Murtezaoglu & Gucer over 102 surgical specimens, it showed that 50% of cases of PTC and none of other thyroid lesions were considered TROP-2 positive with a *p*-value ˂0.05, the much lower level of positivity in both groups is due to different manufacturer, being a polyclonal antibody and shorter incubation period of only 15 min [29].

In the Nesreen et al. study conducted over 110 thyroid samples, TROP-2 showed positive expression in 85.1% of cases of PTC and in 5.6% of other thyroid lesions group with a *p*-value ˂0.05. These results were very similar to our study results due to similar manufacturer of primary antibody [25].

In a study conducted by Yang et al. over 59 surgical specimens, it showed a relatively close result as 96.5% of cases of PTC and 12.5% of other thyroid lesions were considered TROP-2 positive with a *p*-value ˂0.05 [17].

In a study performed by Abdou et al. of 77 thyroid specimens, TROP-2 was positive in 71.4% of PTC cases and in 19% of other thyroid lesions with a *p*-value of ˂0.05 [20]. In Raouf et al. study over 71 thyroid cases, TROP-2 was positive in 90.6% of PTC cases and in 2.6% of other thyroid lesions with a *p*-value of ˂0.001 [30], the manufacture of primary antibody is similar to our study, yet with different cut-off point for positivity.

In Saffar et al. study conducted up on 155 cases of thyroid cases, TROP-2 was positive in 97.6% of PTC cases and in 6.7% of other thyroid lesions with a *p*-value of ˂0.05 [31]. In Attia et al. study conducted up on 75 cases of thyroid cases, TROP-2 was positive in 97.1% of PTC cases and in 5% of other thyroid lesions with a *p*-value of ˂0.05 [23]. These minor variations in levels of expression are due to different sample sizes and cut off point for positivity of TROP-2.

In our study, the calculated sensitivity is 82.28% and the calculated specificity is 92.31%. The calculated positive predictive value is 92.86%, the negative predictive value is 81.08% and the calculated accuracy is 86.81%.

These results of higher specificity and positive predictive value and slightly lower sensitivity and negative predictive value were in line with studies of Addati et al. (2015), Nesreen et al. (2018), Abdou et al. (2019) and Raouf et al. (2020), yet there are different levels of these parameters due to different sample sizes, different composition of control groups, different cut-off points for positivity and different primary antibody manufacturers.

The study conducted by Addati at al. TROP-2 shows sensitivity of 87% and specificity of 89% [26]. TROP-2 in the study performed by Nesreen et al. shows sensitivity of 85.1%, specificity of 94.4%, positive predictive value of 96.9%, negative predictive value of 75.6% and diagnostic accuracy of 88.1% [25].

TROP-2 in the study performed by Raouf et al. shows sensitivity of 90.6%, specificity of 97.4%, positive predictive value of 96.7%, negative predictive value of 92.7% and diagnostic accuracy of 94.4% [30]. The study conducted by Abdou et al. TROP-2 shows sensitivity of 71%, specificity of 81%, positive predictive value of 91%, negative predictive value of 52% and diagnostic accuracy of 74% [20].

On the other hand, studies performed by Yang et al., Zargari and Saffar showed higher sensitivity and negative predictive value and lower specificity and positive predictive value. These controversial results could be related to different sample sizes, different primary antibody producers, different methods of interpretation and different composition of control groups [17, 31, 32].

Yang et al. study showed sensitivity of 96%, specificity of 87.5%, positive predictive value of 88.9%, negative predictive value of 95.5% and diagnostic accuracy of 91.8% [17]. The study conducted by Zargari et al. TROP-2 shows sensitivity of 93%, specificity of 74%, positive predictive value of 81%, negative predictive value of 90% [32]. TROP-2 in the study performed by Saffar et al. shows sensitivity of 98%, specificity of 93%, positive predictive value of 94%, negative predictive value of 97% [31].

In this study there was no statistically significant association between the gender or age of cases in the PTC group and the expression of TROP-2. Also, this study concluded that there is no significant association between the gender and age of cases of other thyroid lesions and the expression of TROP-2. Similar results were concluded by other studies [17, 20, 23, 27]. Yet, there is a statistically significant association between the mean proportion of TROP-2 expression and older age cases (*p* = 0.01).

Also, there was no statistically significant association between all histopathological parameters such as perineural invasion status, lymphovascular, thyroid capsular invasion, extra-thyroidal extension, T stage, lymph nodal metastasis or tumor stage and the expression of TROP-2 either in PTC group or other thyroid malignant tumors group.

Similar results were concluded by studies performed by Bychkov et al. (2016), Yang et al. (2018) and Attia et al. (2024) showing no statistically significant association between all histopathological parameters and expression of TROP-2 in PTC cases and other thyroid lesions. Since, the three researchers have adopted interpretation criteria similar to our research.

On the other hand, the results of Abdou et al. study stated that there is a statistically significant association with presence of lymph nodal metastasis and TROP-2 expression, yet al.l other histopathological parameters have not shown a similar statistically significant association [20].

Yet, in this study there is a statistically significant association between the mean proportion of TROP-2 in tumor cells, older age cases and stage II cases (*p* = 0.02 & 0.02 respectively).

In our study, by combining the results of SLP-2 and TROP-2 and interpreting the results as positive when there is a concomitant SLP-2 and TROP-2 positivity. The sensitivity of combined markers is 79.75%, specificity is 98.46%, positive predictive value is 98.44, the negative predictive value is 80% and the test accuracy is raised to 88.19.

This method of interpretation of combined results raised the test specificity, positive predictive value and the test accuracy in comparison to each single marker. Yet, to the best of our knowledge no other available studies have used a similar interpretation method.

When interpreting the results as positive when there is either SLP-2 or TROP-2 positivity. The sensitivity of combined markers is 93.7%, specificity is 55.4%, positive predictive value is 71.8, the negative predictive value is 87.8% and the test accuracy has decreased to 76.39.

A study conducted by Yang et al. by combining the results of SLP-2 and TROP-2 and interpreting the results as positive when there is either SLP-2 or TROP-2 positivity. Also, there was a higher sensitivity and negative predictive value, lower specificity and positive predictive value and the diagnostic accuracy have not improved. The results of combined markers are 96% sensitivity, 79.2% specificity, 82.8% positive predictive value, 95% negative predictive value and the test accuracy was raised to 87.8% [17].

Also, in this study depending on only the proportion of stained cells by SLP-2 and TROP-2, the diagnostic performance of both markers was calculated with area under the curve of 77.78 and 0.844 for SLP-2 and TROP-2 respectively. The suggested cut off point for discrimination between PTC and other thyroid lesions is > 30% and > 10% for SLP-2 and TROP-2 respectively. These points were not addressed in other research.

## Summary

SLP-2 was positive in 91.1% of PTC samples. Also, 38.5% of other thyroid lesions were positive for SLP-2. Cases of PTC show a statistically significant association with positive SLP-2 expression (p-Value ˂0.001). SLP-2 calculated sensitivity is 91.14% and the calculated specificity is 61.54%. The calculated positive predictive value is 74.23%, the negative predictive value is 85.11% and the calculated accuracy is 77.78%.

There was no statistically significant association between all demographic and histopathological parameters such as gender, age, perineural invasion status, lymphovascular, thyroid capsular invasion, extrathyroidal extension, tumor size, lymph nodal metastasis and tumor stage and the expression of SLP-2 either in PTC group or other thyroid lesions group.

TROP-2 was positive in 82.3% of PTC samples and 7.7% of other thyroid lesions were positive for TROP-2. Cases of PTC show a statistically significant association with positive TROP-2 expression (p-Value ˂0.001).

TROP-2 calculated sensitivity is 82.28% and the calculated specificity is 92.31%. The calculated positive predictive value is 92.86%, the negative predictive value is 81.08% and the calculated accuracy is 86.81%.

There was no statistically significant association between all demographic and histopathological parameters such as gender, age, perineural invasion status, lymphovascular, thyroid capsular invasion, extrathyroidal extension, tumor size, lymph nodal metastasis and tumor stage and the expression of TROP-2 either in PTC group or other thyroid lesions group.

Combining the results of SLP-2 and TROP-2 expressions have been done and the results have been interpreted as positive when there is either SLP-2 or TROP-2 positivity. The sensitivity of combined markers is 93.7%, specificity is 55.4%, positive predictive value is 71.8, the negative predictive value is 87.8% and the test accuracy has decreased to 76.39.

While by interpreting the results as positive when there is a concomitant SLP-2 and TROP-2 positivity. The sensitivity of combined markers is 79.75%, specificity is 98.46%, positive predictive value is 98.44, the negative predictive value is 80% and the test accuracy is raised to 88.19.

## Data Availability

All data generated or analyzed during this study are included in this article.
